# Comparative Determination of Phenolic Compounds in *Arabidopsis thaliana* Leaf Powder under Distinct Stress Conditions Using Fourier-Transform Infrared (FT-IR) and Near-Infrared (FT-NIR) Spectroscopy

**DOI:** 10.3390/plants11070836

**Published:** 2022-03-22

**Authors:** Rahul Joshi, Ramaraj Sathasivam, Praveen Kumar Jayapal, Ajay Kumar Patel, Bao Van Nguyen, Mohammad Akbar Faqeerzada, Sang Un Park, Seung Hyun Lee, Moon S. Kim, Insuck Baek, Byoung-Kwan Cho

**Affiliations:** 1 Department of Biosystems Machinery Engineering, College of Agricultural and Life Science, Chungnam National University, Daejeon 34134, Korea; rahul.joshi98@yahoo.com (R.J.); jpraveenkumar5288@gmail.com (P.K.J.); ajaypatel.iitb@gmail.com (A.K.P.); akbar.faqeerzada@gmail.com (M.A.F.); seunglee2@cnu.ac.kr (S.H.L.); 2 Department of Crop Science, Chungnam National University, 99 Daehak-ro, Yuseong-gu, Daejeon 34134, Korea; ramarajbiotech@gmail.com (R.S.); nguyenvanbao@tuaf.edu.vn (B.V.N.); supark@cnu.ac.kr (S.U.P.); 3 Department of Smart Agriculture Systems, College of Agricultural and Life Science, Chungnam National University, 99 Daehak-ro, Yuseong-gu, Daejeon 34134, Korea; 4 Environmental Microbial and Food Safety Laboratory, Agricultural Research Service, United States Department of Agriculture, Powder Mill Road, BARC-East, Bldg 303, Beltsville, MD 20705, USA; moon.kim@usda.gov (M.S.K.); insuck.baek@usda.gov (I.B.)

**Keywords:** *Arabidopsis thaliana*, phenolic compounds, Fourier-transform IR and NIR spectroscopy, non-destructive

## Abstract

The increasing interest in plant phenolic compounds in the past few years has become necessary because of their several important physicochemical properties. Thus, their identification through non-destructive methods has become crucial. This study carried out comparative non-destructive measurements of *Arabidopsis thaliana* leaf powder sample phenolic compounds using Fourier-transform infrared and near-infrared spectroscopic techniques under six distinct stress conditions. The prediction analysis of 600 leaf powder samples under different stress conditions (LED lights and drought) was performed using PLSR, PCR, and NAS-based HLA/GO regression analysis methods. The results obtained through FT-NIR spectroscopy yielded the highest correlation coefficient ($R_{p}^{2}$) value of 0.999, with a minimum error (RMSEP) value of 0.003 mg/g, based on the PLSR model using the MSC preprocessing method, which was slightly better than the correlation coefficient ($R_{p}^{2}$) value of 0.980 with an error (RMSEP) value of 0.055 mg/g for FT-IR spectroscopy. Additionally, beta coefficient plots present spectral differences and the identification of important spectral signatures sensitive to the phenolic compounds in the measured powdered samples. Thus, the obtained results demonstrated that FT-NIR spectroscopy combined with partial least squares regression (PLSR) and suitable preprocessing method has a solid potential for non-destructively predicting phenolic compounds in *Arabidopsis thaliana* leaf powder samples.

## 1. Introduction

Plants are a great source of various chemical constituents responsible for multiple effects through physical and chemical changes. Some of the most critical components present in plants are phenolic compounds. Plants’ phenolic compounds are defined as specialized metabolites, synthesized through combinations of the shikimate, polyketide, pentose phosphate, and mevalonate pathways. Their chemical structure consists of an aromatic ring with one or more hydroxyl groups [1]. Depending on the number of phenol rings present in the molecule, they can be divided into categories such as phenols, phenolic acids, flavonoids, and isoflavonoids. Phenolic compounds synthesized by plants provide defensive effects by protecting them from UV radiation, unwanted fungi, and pathogens [2,3]. In the past, there was increased interest in determining the phenolic compounds in plants due to their important chemical properties such as antioxidant [4], anti-inflammatory [5], and anticancer activities [6]. 

*Arabidopsis thaliana*, a plant belonging to the mustard family, is well known in plant research science and has thus been renamed as a model plant. This plant plays a critical role in biotechnology due to its various unique properties, i.e., small genome size, easy cultivation, shorter generation time, and high level of seed production, which separate this plant from others [7]. At present, the measurements of phenolic compounds in plants can be carried out by several methods, such as high-performance liquid chromatography (HPLC) [8], gas chromatography (GC), or a combination of GC with mass spectrometry (MS) [9]. Another widely used method for determining a plant’s total phenol/polyphenol content is the Folin–Ciocalteu assay (FC). Blainsk [10] utilized the FC method to determine the total phenolic content from Limonium Brasiliense L. The methods mentioned above are precise, efficient, and provide rapid measurements of the phenolic compounds in plants. However, the applications of these techniques are complicated, often time-consuming, destructive, and generate several chemical waste products, limiting their implementation in real-time applications. Thus, there is an urgent requirement to develop fast, non-destructive, and profitable techniques that can replace these conventional destructive methods for phenolic compound measurements, and further be applied to various plant powder matrices.

Spectroscopic techniques are promising and vital tools for examining the structures of chemically related systems. Specifically, they can help extract a molecule’s structural and physicochemical properties by exploiting the matter and light interactions. They can determine the atomic and molecular structures, and some can even measure the energy differences between various molecular energy levels. Different spectroscopic techniques are available, such as Raman spectroscopy, fluorescence spectroscopy, NMR spectroscopy, etc., which operate under different spectral intervals for measuring experimental parameters, such as the energy of the radiation absorbed or emitted by the molecules and the intensities of the spectral lines [11]. For example, Raman spectroscopy is a vibrational spectroscopy technique that measures the intensity of scattered radiation by absorbing the light radiation of a particular intensity. Furthermore, fluorescence spectroscopy enables the examination of excited electrons present at different energy levels and the color of emitted light after returning to the ground state by the absorption of UV radiation. Additionally, NMR measures the frequencies of the nuclei of some atoms which resonate under a strong magnetic field by the absorption of radiofrequency waves.

Fourier-transform infrared (FT-IR) and Fourier-transform near-infrared (FT-NIR) are key vibrational spectroscopic techniques that measure fundamental vibrations, overtones, and combinations of bands. Both spectroscopic methods offer several advantages over conventional methods by providing fast, non-destructive measurements with little or no sample preparation required. Various researchers have investigated the potential of FT-NIR and FT-IR spectroscopic techniques to perform qualitative and quantitative measurements in different food and agricultural products, i.e., benzene in edible oils [12], glycerol in wines [13], etc.

In the past, multiple studies were performed that have identified the phenolic compounds present in different agricultural products like yerba mate [14], Ginkgo biloba leaf [15], and Radix Salvia Miltrorrhiza extract [16] using spectroscopic techniques. Moreover, in 2020, 2021, and 2022, Arslan et al. [17], Hssaini et al. [18], and Joshi et al. [11] predicted the phenolic compounds in puffs, figs, and moringa powder using a hand-held spectral analytical system and mid-IR spectroscopy in combination with regression analysis. However, the combined applications of FT-IR and FT-NIR spectroscopy for the non-destructive prediction of phenolic compounds in plants under different stress conditions are still limited and not yet fully explored in scientific research. 

Even though FT-IR and FT-NIR spectroscopy offer several advantages, as expressed above, some further drawbacks are associated with both the spectroscopic techniques, i.e., FT-IR spectra are highly affected by the moisture present in samples and have less penetration ability. On the other hand, the generation of overtones and combination bands in FT-NIR spectroscopy results in spectra broadening and makes it less sensitive [11]. Thus, the application of multivariate analysis methods is essential to cope with the limitations of both spectroscopic techniques.

The present study is designed based on the two objectives: (a) to perform a comparative identification using FT-IR and FT-NIR spectroscopy for the non-destructive evaluation of phenolic compounds in *A. thaliana* under various stress conditions (LED lights, drought, or a combination of both), i.e., red+ blue drought, red+ blue non-drought, red drought, red-blue non-drought, white drought, and white non-drought stress conditions; (b) to demonstrate the potential of multivariate analysis methods for classifying powder samples under different stress conditions and predicting the total phenolic compounds in Arabidopsis powder samples.

## 2. Results and Discussion

### 2.1. Spectral Interpretation of Fourier-Transform Infrared (FT-IR) and Fourier-Transform Near-Infrared (FT-NIR)

**Figure 1:**
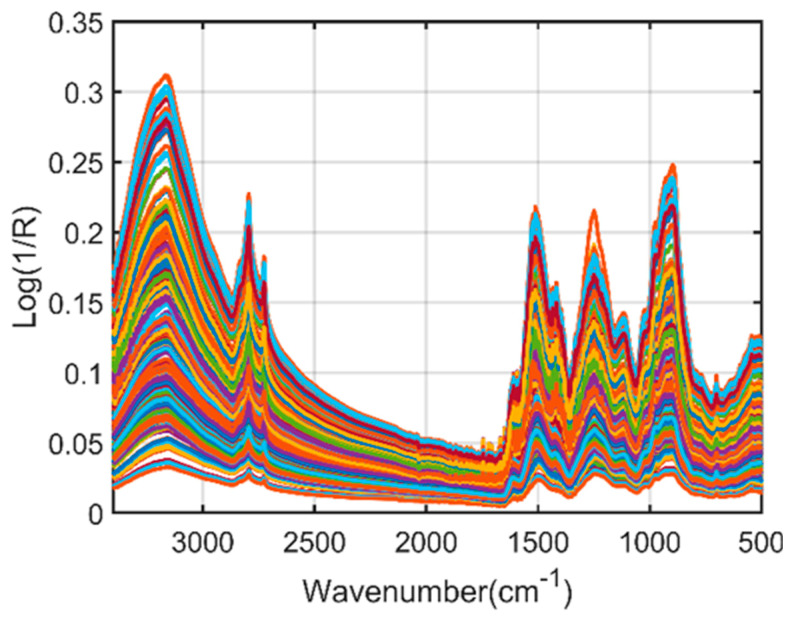
Fourier-transform infrared (FT−IR) raw spectra of *A. thaliana* powder samples.

Figure 1 shows the raw spectra of *Arabidopsis thaliana* powder samples acquired through an FT-IR spectrophotometer. The raw spectra usually consist of overlapping peaks due to the noise generated through external factors. This suppressed the information essential for identifying the phenolic compounds in the scanned samples. Spectral preprocessing is necessary to obtain high-quality data by removing the external noise and overlapping peaks. 

**Figure 2:**
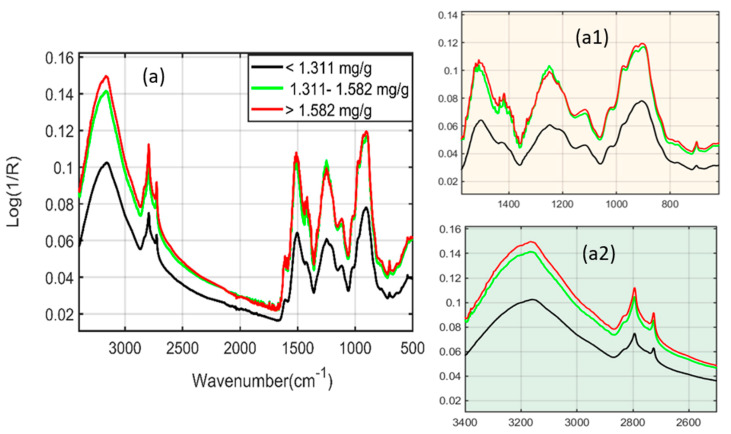
Standard normal variate (SNV) preprocessed FT−IR spectra (**a**). (**a1**,**a2**) are the extended spectral regions related to phenolic compounds.

Figure 2a presents the SNV-preprocessed spectra of the scanned plant powder samples under six different stress conditions. The six different concentration values obtained through the reference HPLC analysis method were divided into three categories, i.e., <1.311 mg/g, 1.311–1.582 mg/g, and >1.582 mg/g. The FT-IR spectra were classified into two essential regions, i.e., the functional group and fingerprint regions. The functional group region (FGR) was observed at around 4000–1450 cm^−1^, whereas the fingerprint region (FPR) ranged from 1450 to 500 cm^−1^. Both spectral areas had unique characteristics, i.e., the FGR typically corresponded to the stretching vibrations of atoms and molecules, therefore resulting in fewer peaks. On the other hand, FPR is considered a highly informative region because each compound has unique spectral signatures, resulting in more spectral peaks. There was no relevant information below 500 cm^−1^ and above 3600 cm^−1^; therefore, the spectra were plotted between wavelengths of 3600 and 500 cm^−1^, respectively. The spectral signatures observed between 3500 and 2500 cm^−1^ and 1700 and 1600 cm^−1^ represent the stretching vibrations of the hydroxyl (OH) and carbonyl (C=O) functional groups present in the phenolic compounds of Arabidopsis samples, respectively. All six different concentrations were differentiated from each other within this range, as shown in Figure 2a1,a2. Phenolic compounds consist of a phenolic benzene ring (C_6_H_5_OH) and different functional groups such as OH, C=O, and C-H; thus, they are responsible for generating stretching and bending vibrations. The vibrations obtained around 2954 and 2850 cm^−1^, 1505 cm^−1^, 1600 cm^−1^, and 1505 cm^−1^ are related to C-H stretching [19], C=C stretching, benzene ring skeleton [20], and C=C aromatic stretching [21], as explained in Table 1.

**Figure 3:**
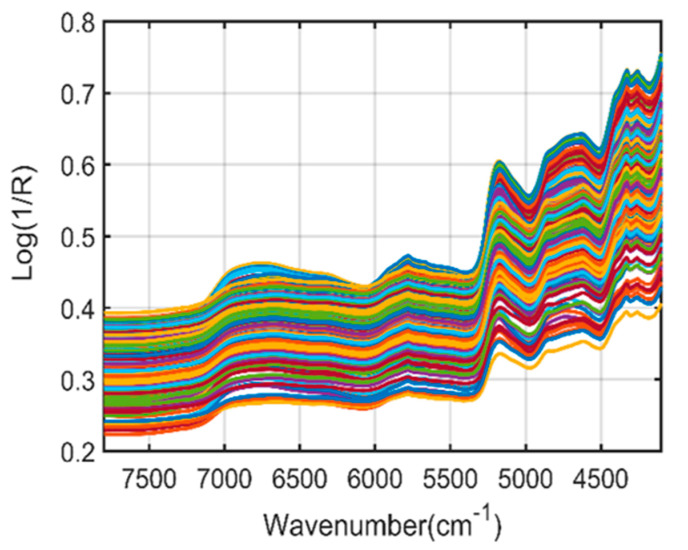
Fourier-transform near-infrared (FT−NIR) raw spectra of *A. thaliana* powder samples.

Figure 3 represents the original FT-NIR spectra of *Arabidopsis* powder samples from the 4000–10,000 cm^−1^ wavenumber range. Since the raw spectra do not provide clear information due to the presence of overlapping peaks and noise, preprocessing steps were further performed to enhance the spectral quality and acquire meaningful information related to the phenolic compounds of the *A. thaliana* powder samples.

**Figure 4:**
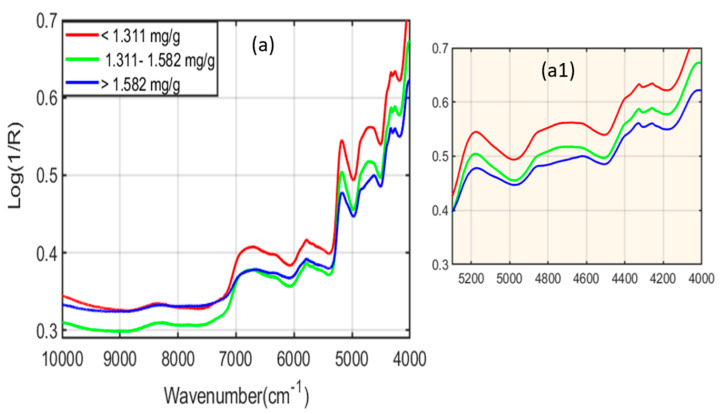
FT−NIR SNV preprocessed spectra (**a**). (**a1**) is the extended spectral regions related to phenolic compounds.

The multiplicative scattering correction (MSC) preprocessed spectra shown in Figure 4a exhibit significant spectral peaks for all six different phenolic concentrations ranging from <1.311 mg/g to 1.311–1.582 mg/g and >1.582 mg/g, which were identified through the HPLC method. Various important characteristic peaks for the phenolic compounds were observed in the preprocessed spectra in Figure 4a and the extended regions (a1) around 8350 cm^−1^, 6000–7000 cm^−1^, and 5500–6000 cm^−1^, which are associated with the second overtone of C-H stretching, the first overtone of the O-H and N-H stretching [22], and the first overtone of C-H stretching vibrations [23], respectively. 

Moreover, additional peaks were also observed from 4450 to 4285 cm^−1^, 4450 to 4410 cm^−1^, 4380 to 4315 cm^−1^, and 4285 cm^−1^, which were responsible for the combination band regions. The O-H bond combined with the C-O bond and the C-H bond, and the C-H bond combined with the C-H bond [23], which are subsequently sensitive to phenolic compounds present in the *A. thaliana* powder samples, as elaborated in Table 2. 

### 2.2. Dirichlet Distribution

**Figure 5:**
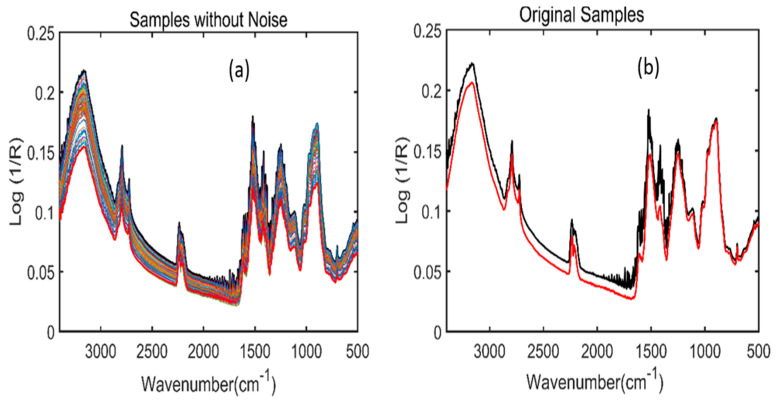
A total of 100 mixed samples FT−IR spectra of Arabidopsis powder samples for one concentration created by Dirichlet distribution (**a**). FT-IR spectra developed between two replicates, i.e., red drought_1 and red non-drought_1 (RD_1 and RND_20) for one variety of concentrations (**b**).

**Figure 6:**
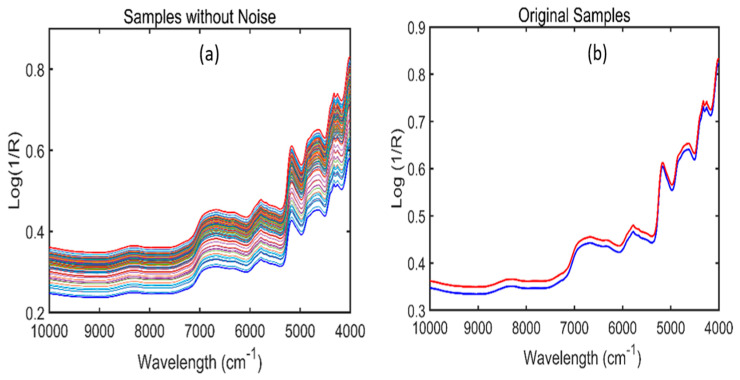
A total of 100 mixed samples FT-NIR spectra of Arabidopsis powder samples for one concentration created by Dirichlet distribution (**a**). FT-NIR spectra developed between two replicates i.e., white drought_1 and white non-drought_1 (WD_1 and WND_10) for one concentration (**b**).

During FT-IR and FT-NIR spectral acquisition, only ninety sample spectra were acquired for each of the six different phenolic concentrations under various stress conditions (LED lights + water), which is not enough to develop a strong multivariate analysis model. Due to presence of a fewer number of samples, the phenomena of underfitting occurs. In order to solve this problem, an algorithm introduced by Dirichlet was utilized in this study to avoid the underfitting issue. The detailed mathematical explanation of this algorithm is presented elsewhere [24]. The Dirichlet distribution algorithm generated 600 synthetic data for each spectroscopic technique which were later used for model development. The working procedure of this algorithm has been symbolized through Figure 5 and Figure 6. Here, the term sample without the noise in Figure 5 and Figure 6 represents the preprocessed spectra after the generation of synthetic spectral data, while the original data term in Figure 5 and Figure 6b represent the original spectra of RD_1 and RD_20 and WD_1 and WD_10 respectively. 

### 2.3. Principal Component Analysis (PCA) of Samples under Different Stress Conditions

**Figure 7:**
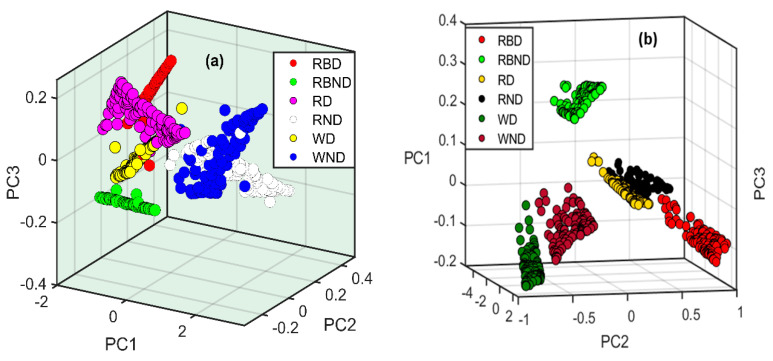
Principal component analysis of Arabidopsis thaliana powder samples for (**a**) FT−IR and (**b**) FT-NIR spectroscopy under different stress conditions. Here, the abbreviations RBD, RBND, RD, RND, WD, and WND stand for red+ blue drought, red+ blue non-drought, red drought, red-blue non-drought, white drought, and white non-drought.

Principal component analysis is a widely used unsupervised method for visualizing data by performing dimension reductions in machine learning. First, PCA was applied to the preprocessed data of the Arabidopsis thaliana powder samples to check its ability to differentiate the samples under different stress conditions. Figure 7a presents the resultant 3D scatter plot for the FT-IR spectroscopic data, showing clustering among the samples depending upon the changes in phenolic concentration. The first three principal components (PCs), i.e., PC1, PC2, and PC3, accounts for highest present in the spectral data, which are nearly 95%. In contrast, the remaining PCs did not show significant changes in the variance, and mainly presented noise in the data. For the FT-IR spectroscopic data, the PCA model was not capable of differentiating between the samples under six different stress conditions and resulted in the overlapping of spectral data due to the reduced sensitivity of the FT-IR towards the Arabidopsis powder samples. The 3D PCA scatter plot for the FT-NIR spectra is shown in Figure 7b, showing clear sample discrimination under different stress conditions. The first three PCs exhibit maximum variance in the sample data, whereas the remaining PCs are less informative. The developed PCA model completely isolated all six conditions, whereas RD and RND data slightly overlapped due to less difference between the phenolic concentrations under these conditions. Thus, it is suggested that PCA provides more vital support to FT-NIR spectroscopy data than FT-IR for the clear visualization and further discrimination of plant powder samples under various stress conditions.

### 2.4. High Performance Liquid Chromatography (HPLC) Reference Analysis for the Phenolic Compound Measurements

The reference HPLC values acquired for phenolic compounds in *A. thaliana* powder samples are detailed in Table 3. The analysis was performed for 90 plant samples that were grown under different light and water conditions (drought and non-drought). The mean value of the three biological replicates were statistically analyzed by analysis of variance (ANOVA) with Duncan’s multiple range test (DMRT) set to *p* < 0.05 used for the data analysis. This was done using SAS software version 9.2 (SAS Institute Inc., Cary, NC, USA, 2009).

### 2.5. PLSR, PCR, and HLA/GO Prediction Results for FT-IR and FT-NIR Spectroscopy

After the generation of the artificially mixed 600 samples using the Dirichlet algorithm, the regression analysis models were constructed by creating calibration and prediction datasets. For the calibration dataset, 360 samples (60 samples from each condition) were used out of 600 samples. In comparison, the remaining samples for the prediction dataset consisted of 240 samples (40 samples from each condition) shown in Table 4. Three different regression analysis methods, namely, PLSR, PCR, and HLA/GO, were employed for the non-destructive estimation of phenolic compounds in the scanned powdered samples.

#### 2.5.1. Prediction Analysis Results of FT-IR Spectroscopy

**Figure 8:**
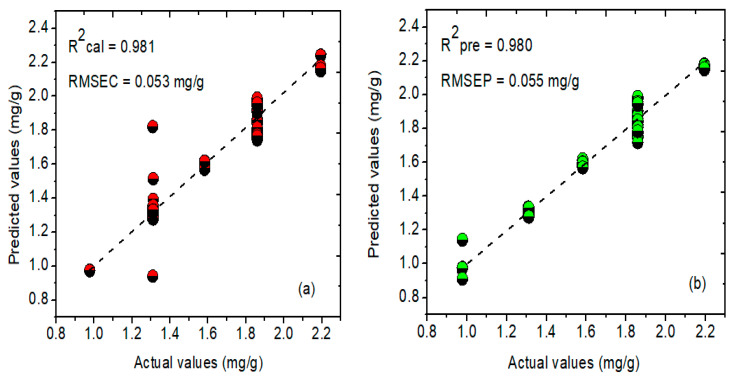
Actual and predicted concentration values for the phenolic compounds in *A. thaliana* leaf powder samples using the PLSR model for (**a**) calibration and (**b**) prediction datasets. Here, RMSEC and RMSEP represent root-mean-square error for calibration and prediction.

Firstly, the partial least squares (PLSR) model was developed to perform the prediction analysis of phenolic compounds. During the model development, different preprocessing steps, such as normalization, MSC, SNV, and Savitzky–Golay derivatives (first and second) were used, out of which SNV bears a higher correlation coefficient (R^2^) value of 0.981, with a minimum root-mean-square error (RMSEC) value of 0.053 for the calibration dataset. On the other hand, the R^2^ and RMSEP values acquired for the prediction dataset were 0.980 and 0.055 mg/g, respectively. Figure 8a,b depicts the actual and predicted values derived through the PLSR model, clearly showing a good relationship between the two groups.

**Figure 9:**
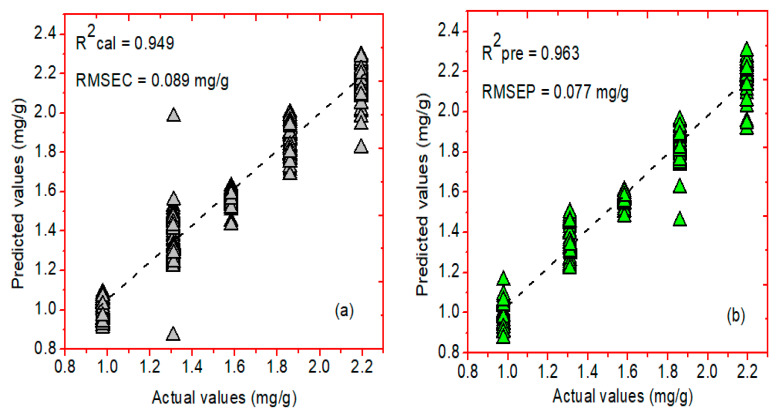
PCR graphs of actual and predicted concentration values for the phenolic compounds in Arabidopsis powder samples using (**a**) calibration and (**b**) prediction datasets, respectively.

To compare the prediction ability of the developed PLSR model, two different regression methods, i.e., PCR and NAS-based HLA/GO, were chosen. Figure 9a,b symbolizes the relationship between actual and predicted concentrations of the phenolic compounds determined through the PCR model using the Savitzky–Golay first derivative preprocessing method. The PCR model developed for FT-IR spectral data attained a correlation coefficient (R^2^) value of 0.949, and a root-mean-square error of (RMSEC) value of 0.089 mg/g for the calibration dataset, while the R^2^ and RMSEP values for the prediction dataset were 0.963 and 0.077 mg/g, respectively.

**Figure 10:**
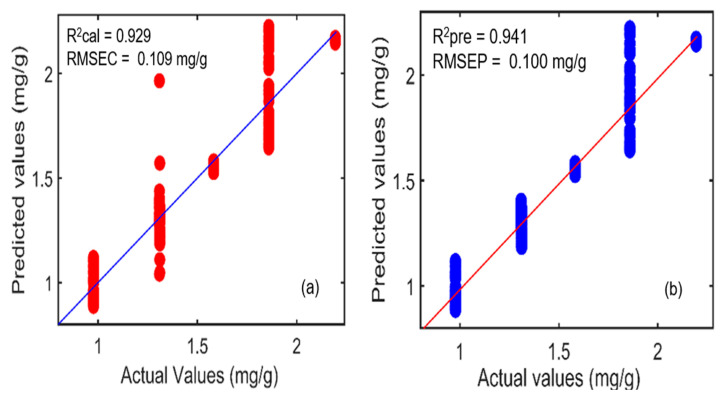
Hybrid linear analysis (HLA/GO) model for actual and predicted concentration values for the phenolic compounds in *A. thaliana* powder samples for (**a**) calibration and (**b**) prediction datasets.

Furthermore, in the same manner, the NAS-based hybrid linear analysis (HLA/GO) model was established using the SNV pretreatment method. The correlation coefficient (R^2^) values acquired for the calibration and prediction datasets were 0.929 and 0.941, respectively, whereas the error values (RMSEC and RMSEP) were 0.109 mg/g and 0.100 mg/g, respectively. Figure 10a,b symbolizes the relationship between the actual and predicted concentrations of phenolic compounds determined through the NAS-based HLA/GO model using the SNV preprocessing method. Table 5 presents the prediction results acquired through all three regression methods. The results specified that the developed PLSR model performed better than the PCR and HLA/GO models, with a higher correlation coefficient (R^2^) value of 0.980 and the lowest root-mean-square error (RMSEP) value of 0.055 mg/g for the prediction dataset. 

#### 2.5.2. Prediction Results for FT-NIR Spectroscopy

**Figure 11:**
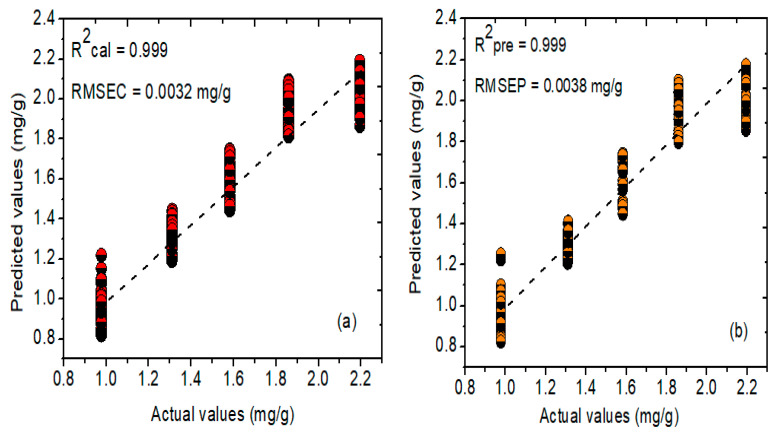
Actual and predicted concentration values for the phenolic compounds in *A. thaliana* leaf powder samples using the PLSR model for (**a**) calibration and (**b**) prediction datasets. Here, RMSEC and RMSEP represent the root-mean-square error for calibration and prediction, respectively.

For the FT-NIR spectral data, first, the PLSR models were constructed using different preprocessing methods. In comparison with other preprocessing methods, i.e., normalization, MSC, SNV, and Savitzky–Golay derivatives (first and second), MSC performed slightly better and acquired higher calibration and prediction correlation coefficient (R^2^) values of 0.999 with minimum error values (RMSE) of 0.003 mg/g, respectively. Figure 11a,b depicts the actual and predicted values derived through the PLSR model, which clearly shows a good relationship between the two groups. 

**Figure 12:**
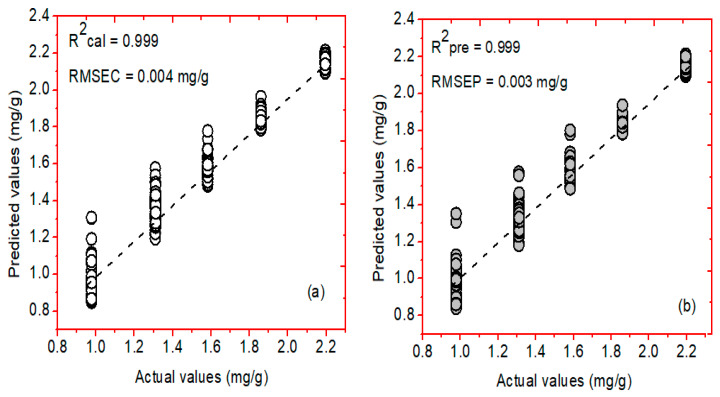
PCR graphs of actual and predicted concentration values for the phenolic compounds in *A. thaliana* leaf powder samples using (**a**) calibration and (**b**) prediction datasets, respectively.

On the other hand, the PCR and NAS-based HLA/GO models were further developed to compare the prediction performance and acquire the best prediction model using a similar number of samples chosen during PLSR model development. Figure 12a,b symbolizes the relationship between actual and predicted concentrations of phenolic compounds determined through the PCR model using the Savitzky–Golay first derivative preprocessing method. The PCR model developed for the FT-NIR spectral data attained a correlation coefficient (R^2^) value of 0.999 and a root-mean-square error of (RMSEC) value of 0.004 mg/g for the calibration dataset. In comparison, the R^2^ and RMSEP values for the prediction dataset were 0.999 and 0.003 mg/g, respectively. 

**Figure 13:**
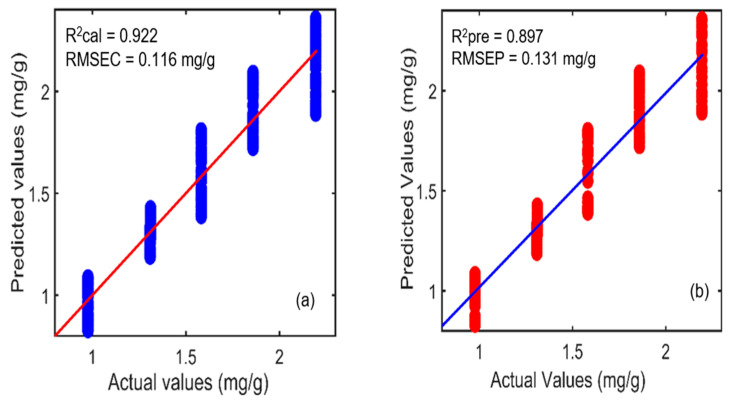
Actual and predicted concentration values for the phenolic compounds in *A. thaliana* leaf powder samples using the hybrid linear analysis (HLA/GO) model for (**a**) calibration and (**b**) prediction datasets. Here, RMSEC and RMSEP represent the root-mean-square error for calibration and prediction, respectively.

Furthermore, similarly, the NAS-based hybrid linear analysis (HLA/GO) model was established using different preprocessing methods, out of which SNV exhibited better prediction performance results. The correlation coefficient (R^2^) values acquired for the calibration and prediction datasets were 0.929 and 0.897, whereas the error values (RMSEC and RMSEP) were 0.116 mg/g and 0.131 mg/g, respectively. Figure 13a,b depicts the actual and predicted values derived through the NAS-based HLA/GO model, exhibiting the relationship between the two groups. Table 6 presents the prediction results acquired through all three regression methods. Based on the results, it can be stated that the PLSR model performed better than the other two regression methods by achieving a higher correlation coefficient (R^2^) value of 0.999 and a lower root-mean-square error (RMSEP) value of 0.033 mg/g for the prediction dataset. The PCR achieved similar prediction results, although the error value (RMSEC) was slightly higher than the PLSR model for the calibration dataset. Thus, the PLSR model is superior to both of the other models for the required prediction analysis.

### 2.6. Beta Coefficients Results of FT-IR and FT-NIR Spectroscopy

**Figure 14:**
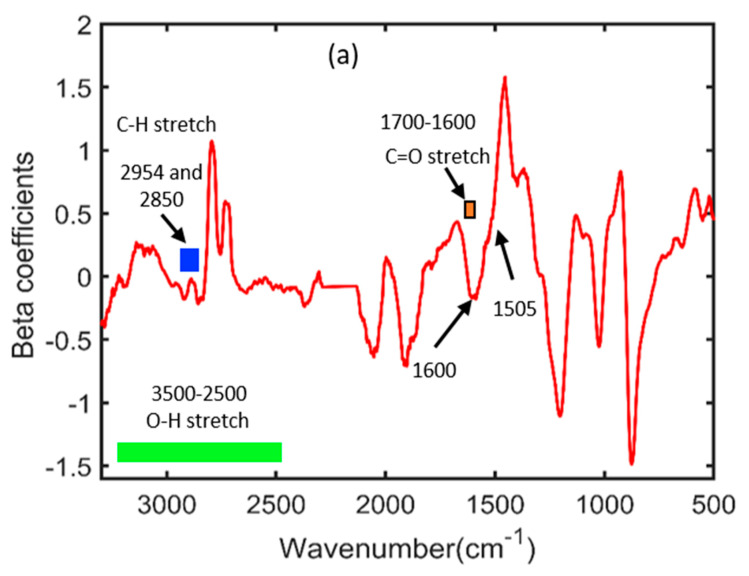

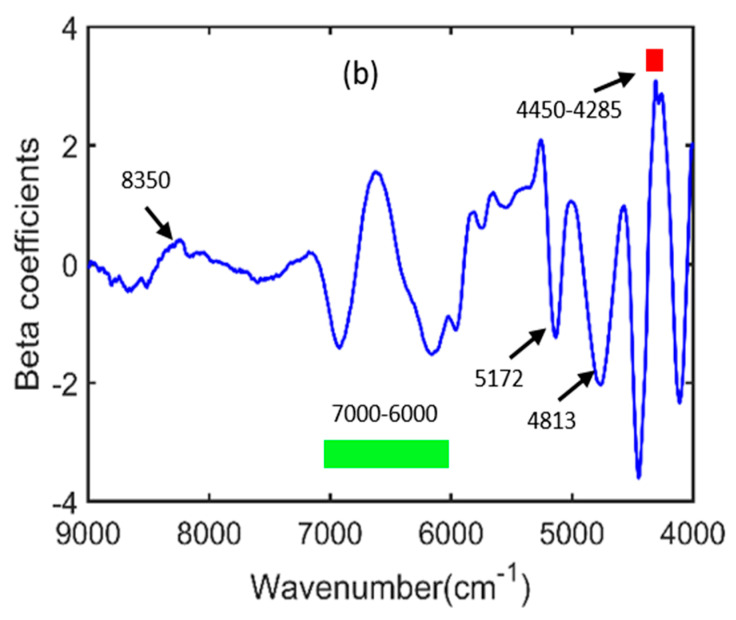
Beta coefficient plots developed through the PLSR model: (**a**) FT−IR spectroscopy and (**b**) FT−NIR spectroscopy.

The results acquired through the PLSR model resulted in better performance for both the spectroscopic techniques; therefore, the beta coefficients were plotted to illustrate significant wavenumbers, which are crucial for providing information regarding phenolic compounds’ chemical structures. The FT-IR spectroscopy beta plot is presented in Figure 14a, which shows essential spectral signatures under a suitable wavelength range. The spectral regions from 3500 to 2500 cm^−1^ and 1700 to 1600 cm^−1^ addressed the O-H and C=O stretching vibrations of phenolic compounds, respectively. Additionally, a few peaks were observed around 1600, 1505, and 1500 cm^−1^, identical to those observed in Figure 2a, indicating the sensitive regions of phenolic compounds identified through FT-IR spectroscopy.

On the other hand, Figure 14b shows the beta coefficient plot for FT-NIR spectroscopy obtained through the MSC preprocessing method. The beta plot developed through FT-NIR spectroscopy depicts the prominent characteristics of spectral peaks related to the phenolic compounds of the Arabidopsis powder samples. The regions from 7000 to 6000 cm^−1^ and 4450 to 4285 cm^−1^ are combination band regions related to the first overtones of O-H and N-H stretching. Moreover, some peaks were also noticed around 8350, 5172, and 4813 cm^−1^, associated with the second overtone of C-H stretching, a combination of O-H and N-H stretching, and a combination of O-H and C-O stretching, respectively; this was identical to the spectral signatures observed in Figure 4a. FT-NIR spectroscopy has a higher penetration depth than FT-IR spectroscopy; thus, it provides more detailed information related to the chemical structures of compounds present in the samples under examination. The prediction analysis results presented in Section 2.5.2. completely supported this statement; hence, it can be suggested that FT-NIR spectroscopy coupled with a partial least square regression (PLSR) method could be an alternative tool for the non-destructive examination of phenolic compounds in *A. thaliana* powder samples. In the previous reports, Claveria [25] measured phenolic compounds in senescent and water-stressed tobacco by using high-performance liquid chromatography coupled to electrospray ionization tandem mass spectrometry, while Villagra [26] measured phenolic compounds in the leaves of Aristotelia chilensis plants (Mol.) subjected to drought stress using HPLC-photodiode array detection. Although these techniques are susceptible and precise for measuring phenolic compounds (flavonoid) and can measure at nanogram and picogram levels, the applications of these techniques are often time-consuming, destructive, and generate much chemical waste. On the other hand, Joshi [11], Arslan [17], and Hssaini [18] performed phenolic compounds identification using MIR spectroscopy and a hand-held spectral analytical system and acquired a higher correlation (R^2^) value of 0.99; still, their studies are limited and cannot provide measurements of phenolic compounds under different stress conditions (LED lights + water). The limitations of all aforementioned studies were resolved in this study by measuring the phenolic compounds of *A. thaliana* plants powder matrices under various stresses and acquiring a higher correlation coefficient (R^2^) value of 0.999 and a minimum error (RMSEP) value of 0.003 mg/g. Further, this research provides fast and easy sample preparation procedures by scanning a large number of samples, which further helped in constructing more robust prediction models. Hence, the results acquired proved that partial least square regression (PLSR) with MSC preprocessing, when conjugated with FT-NIR spectroscopy, can be utilized instead of the destructive chemical methods for the assessment of phenolic compounds in Arabidopsis thaliana powder samples under different stress conditions, and can replace the conventional analytical techniques in a rapid manner.

## 3. Materials and Methods

### 3.1. Sample Preparation

**Figure 15:**
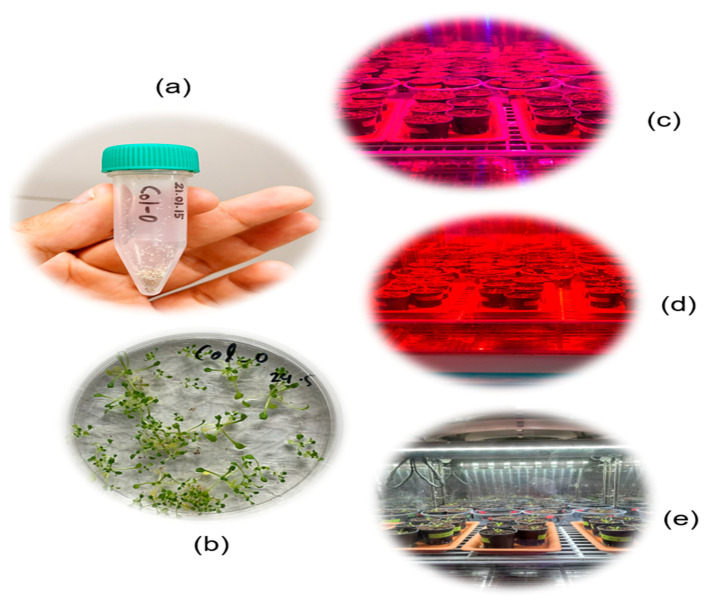
Arabidopsis Col-10 seeds (**a**), seeds grown in a Petri dish (**b**) under different lighting conditions: (**c**) red + blue, (**d**) red, and (**e**) white.

In this study, Arabidopsis thaliana plant samples were used. For the germination of Arabidopsis seeds, the Arabidopsis thaliana “Col-10” seeds shown in Figure 15a were grown on a Petri dish in the presence of a suitable medium (Figure 15b) at 4 °C for 7 days. After the germination was finished, the seedlings were plucked from the Petri dish and carefully moved into the soil pots, ensuring that they did not break. Before the seedlings were replanted in the pots, the soil was autoclaved at 125 °C for 20 min to remove unwanted species such as fungi or germs from the soil. The pots were later relocated to inside a growth chamber under three different LED lighting conditions, such as red+ blue light, red light, and white light, as shown in Figure 15c–e, for 8 h. The plants were grown inside the room at a 25 °C temperature and 70% humidity, conditions which were kept constant throughout the experiment. Drought stress was induced on the plants after two weeks, where 5 mL of water was poured into half of the plants inside the chamber for each lighting condition. 

### 3.2. Plants during Non-Stress and Stress (Drought) Conditions

**Figure 16:**
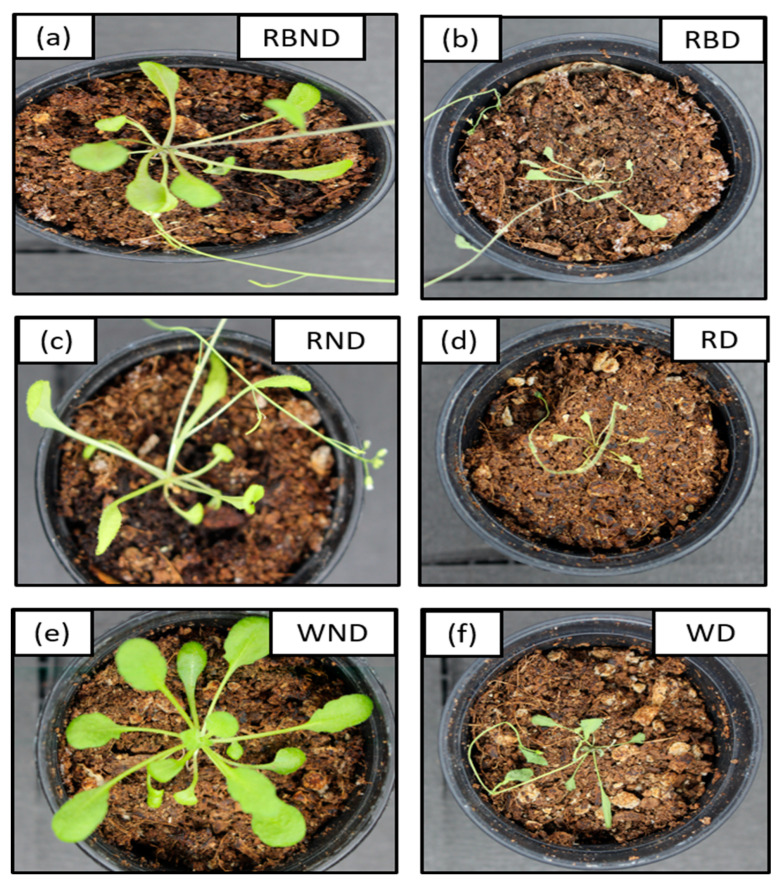
RGB images of non-drought and drought conditions of *A. thaliana* plants under different LED lightning conditions. Here, RBND and RBD (**a**,**b**) represents red + blue non-drought and red + blue drought, RND and RD (**c**,**d**) represents red non-drought and red drought, and WND and WD (**e**,**f**) represents white non-drought and white drought respectively.

The *A. thaliana* plants under six different stress conditions (LED + water) are presented in below Figure 16. 

In this study, in addition to controlled plants, we also used different stress condition plants (LED + water) in order to perform the prediction analysis of phenolic compounds in *A. thaliana*. The plants under white light are considered controlled plants. For two weeks, drought stress was not applied to the plants to grow well, and later, after two weeks, we used drought stress to observe the change in the phenolic compounds, so they are called stressed plants. Based on the above Figure 16, it can be observed clearly that after applying drought stress, the plants’ health conditions significantly decreased. Therefore, it results in a significant change in phenolic compounds, which was later confirmed from the HPLC analysis result shown in Table 3 in Section 2.4.

### 3.3. HPLC Analysis

Due to the small size of Arabidopsis thaliana leaves, and due to high cost of instrumentation, HPLC analysis was carried out for groups rather than measuring them individually. In this study, 90 Arabidopsis thaliana plants were grown inside the growth chamber under 6 different stress conditions (LED lights + water), i.e., red+ blue drought, red+ blue non-drought, red drought, red non-drought, white drought, and white non-drought. For individual conditions, 15 plants were chosen as follows: 90 plants/6 conditions = 15 plants per condition. The phenolic compounds were extracted from Arabidopsis leaves according to the protocol described previously by Yeo et al. [27], with minor modifications. From each condition, 0.1 g of powder leaves sample was taken and mixed with 3 mL of 80% aqueous methyl alcohol (MeOH) solution. Subsequently, the prepared mixture was vortexed for 1 min to make a uniform mixture and was further sonicated (JAC Ultrasonic 4020, Hwaseong, Gyeonggi-Do, Korea) for 1 h at 37 °C. The mixture was then centrifuged (Mega 21 R, Hanil Science Inc., Gimpo, Korea) at 4 °C for 15 min at 10,000 rpm. The clear supernatants obtained after centrifugation were brought together and filtered through a 0.45 µm PTFE syringe filter (Millipore, Bedford, MA, USA) into amber glass vials (Thermo Fisher Scientific, Waltham, MA, USA). The HPLC machine, conditions, mobile phase, column, and gradient program were identical to the protocol described by Yeo et al. [27]. 

### 3.4. FT-IR and FT-NIR Spectroscopy

The FT-IR spectral measurements of *Arabidopsis* powder samples were performed using a laboratory-based Nicolet 6700 (Thermo Scientific Co.) FT-IR spectrometer. The spectrometer was equipped with attenuated total reflectance (ATR) sampling mode. Furthermore, the system consisted of a deuterated triglycine sulfate (DTGS) detector and employed potassium bromide (KBr) as a beam splitter, which were controlled together by the OMINIC software. The spectral acquisition was performed at 4000–400 cm^−1^ wavelengths. During the spectral acquisition, the sample was deposited on the surface of the diamond crystal sampling plate. A total of 32 scans were acquired at 4 cm^−1^ spectral intervals for each sample, and the average spectral data were saved in Excel format for further analyses.

The spectral acquisition of *Arabidopsis* powder samples was performed by adopting a laboratory-based Antaris II FT-NIR analyzer (Thermo Scientific Co., Waltham, MA, USA). The system incorporated an InGas detector which could perform spectral data collection within a wavelength range of 4000–10,000 cm^−1^. The powder samples were deposited on the sample holder and covered with a black lid to avoid fluctuations from the background environment. The spectrometer was operated in reflectance mode, which collected the spectrum for each sample by performing 32 scans at 4 cm^−1^ spectral intervals. The average spectrum was used for the spectral analysis, which was saved in Excel format.

### 3.5. Data Analysis

Undesirable noise, such as instrumental drift, particle size, and background effects generated during FT-IR and FT-NIR spectroscopic data collection, is often responsible for making the data unclean, reducing their effectiveness, and directly impacting a model’s prediction capability. Spectral pretreatment thus plays an essential role in keeping noise away from the acquired spectral data and collecting accurate chemical information. Therefore, the raw spectra first needed to be corrected by applying preprocessing methods. In this research, both FT-IR and FT-NIR spectral data were treated with several different preprocessing methods, namely, mean normalization, range normalization, standard normal variate, multiplicative scatter correction (MSC), standard normal variate (SNV), and Savitzky–Golay (SG) derivatives (1st and 2nd). For a detailed description of the various preprocessing methods, refer to [28].

**Figure 17:**
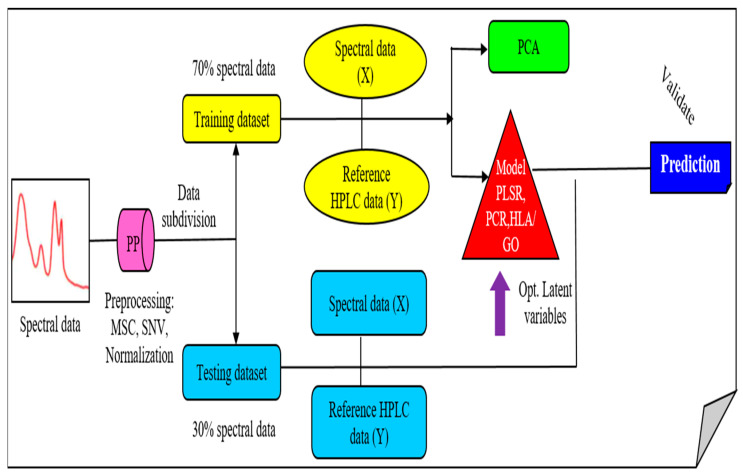
Flowchart for *A. thaliana* powder samples spectral data analysis.

After the preprocessing steps were performed, spectral data of the Arabidopsis powder samples were analyzed using the multivariate analysis method, which includes principal component analysis (PCA), principal component regression (PCR), partial least square regression (PLSR), and support vector regression (SVR). The complete spectral analysis was performed using MATLAB (version 7, MathWorks, Natick, MA, USA). The flowchart of the complete spectral data analysis of *A. thaliana* powder samples shown in Figure 17. 

#### 3.5.1. PCA Model 

For data visualization, and to classify *Arabidopsis thaliana* powder samples under different stress conditions, principal component analysis (PCA), a popular unsupervised multivariate analysis method, was applied to the spectroscopic data. This is a dimensional reduction method that reduces large datasets by transforming more variables into fewer numbers without reducing the information present in the large dataset [11]. The first principal component (PC1) describes the maximum variance in the data, whereas the second principal component (PC2), which is orthogonal to PC1, presents a more minor variance.

#### 3.5.2. Prediction Analysis Models

Partial least square regression (PLSR), principal component regression (PCR), and NAS-based hybrid linear analysis (HLA/GO) were used to predict the phenolic compounds in Arabidopsis powder matrices, and their performance was compared. The PLSR method is primarily used when high collinearity exists among the predicting variables. This technique derives a linear relationship between one of the dependent variables and independent variables. PLSR is one of the most widely used algorithms by researchers to perform prediction analyses for quantitative measurements [29]. The general equations derived for PLSR are as follows:

X = TP^T^ + E (1)

Y = UQ^T^ + E (2)

where the terms X and Y represent independent and dependent variables, respectively, T and U are score matrices for X and Y, P^T^ and Q^T^ denote loading matrices, and E represents the error matrix.

Another widely applicable method for solving multicollinearity issues present in the data is PCR, defined as a combination of principal component analysis (PCA) and multivariate linear regression (MLR). In the first step, PCA is performed, which decomposes the spectral data through Equation (1), and in the next step, the optimum number of principal components acquired through PCA is utilized in the MLR model to carry out PCR [30]. 

Furthermore, NAS regression-based HLA/GO algorithms have also been utilized to predict phenolic compounds in Arabidopsis powder samples. A comprehensive explanation regarding mathematical equations is presented in [31]. In the NAS algorithm, the analyte concentration under investigation is directly proportional to the portion of the signal calculated by NAS [32]. The NAS vector for each sample under study was determined based on the procedure described by Goicoechea and Olivieri [31] and Marsili [33].

## 4. Conclusions

This study was designed to perform the comparative evaluation of phenolic compounds in *Arabidopsis thaliana* powder samples using two different vibrational spectroscopic techniques, i.e., FT-IR and FT-NIR spectroscopy under different stress conditions (either light or drought stress or a combination of both) regarding high-performance liquid chromatography (HPLC), a reference analysis method. PCA was used for the data visualization and to discriminate the powder samples under six different stress conditions (water and light). The prediction analysis of phenolic compounds was done using PLSR, PCR, and NAS-based HLA/GO multivariate analysis methods. Both spectroscopic techniques showed notable results, but FT-NIR performed superior to FT-IR spectroscopy. The results acquired through FT-NIR spectroscopy achieved the highest correlation coefficient ($R_{p}^{2}$) value of 0.999 with the minimum error (RMSEP) value of 0.003 mg/g when using the MSC preprocessing method. They resulted in better performance compared to the HLA/GO and PCR methods. Further, PCA also supports the clear discrimination of all six distinct stress conditions for the FT-NIR spectroscopy than for the FT-IR. The acquired results clearly demonstrate the potential of our developed model. Therefore, we can conclude that FT-NIR spectroscopy in conjugation with partial least squares regression (PLSR) and suitable preprocessing method could replace the conventional destructive analytical techniques and serve as a rapid analytical tool for the non-destructive measurement of phenolic compounds in Arabidopsis thaliana leaf powder samples under different stress conditions. The following research will further proceed for other powder matrices to evaluate the validity of the constructed model in real-world applications.

## Figures and Tables

**Table 1:** FT-IR spectral vibrations of phenolic compounds observed in *A. thaliana* leaf powder samples.

Spectroscopic Technique	Absorption Frequency, ν (cm^−1^)	Assignment
FT-IR spectroscopy	3500–2500	O-H stretching
	1700–1600	C=O stretching
	2954 and 2850	C-H stretching
	1505	C=C stretching
	1600	Benzene ring skeleton
	1500	C=C aromatic stretching

**Table 2:** FT-NIR spectral vibrations of phenolic compounds observed in *A. thaliana* leaf powder samples.

Spectroscopic Technique	Absorption Frequency, ν (cm^−1^)	Assignment
FT-NIR spectroscopy	8350	Second overtone of C-H stretching
	6000–7000	First overtone of the O-H and N-H stretching
	5172	Combination of O-H and C-O stretching
	4813	Combination of O-H bending and C-O stretching
	4450 to 4285	Combination band region
	4450 and 4410	O-H bond combined with the C-O bond
	4380 and 4315	the C-H bond
	4285	The C-H bond combined with the C-H bond

**Table 3:** The reference values of phenolic compounds (mg/g dry weight (DW)) obtained from the HPLC analysis.

Phenolics	Red + Non-Drought	Red + Drought	Red-Blue + Non-Drought	Red-Blue + Drought	White + Non-Drought	White + Drought
Gallic acid	ND	ND	ND	ND	0.043 ± 0.010 a ^1^	0.017 ± 0.002 b
Catechin	0.141 ± 0.003 a	0.165 ± 0.021 a	0.130 ± 0.014 a	0.134 ± 0.014 a	0.150 ± 0.011 a	0.145 ± 0.026 a
Chlorogenic acid	0.123 ± 0.002 a	0.119 ± 0.011 a	ND	ND	0.123 ± 0.005 a	0.137 ± 0.009 a
Caffeic acid	0.049 ± 0.010 b	0.060 ± 0.007 b	ND	ND	0.059 ± 0.005 b	0.050 ± 0.011 b
(-)-Epicatechin	ND	ND	ND	ND	0.055 ± 0.011 b	0.037 ± 0.004 b
Epicatechin gallate	0.124 ± 0.006 c	0.255 ± 0.030 b	ND	ND	0.743 ± 0.023 a	0.302 ± 0.030 b
Ferulic acid	0.033 ± 0.013 cd	0.053 ± 0.001 c	ND	ND	0.138 ± 0.014 b	0.384 ± 0.029 a
Sinapic acid	ND	0.015 ± 0.002 b	ND	ND	0.032 ± 0.002 a	0.035 ± 0.007 a
Benzoic acid	0.136 ± 0.002 b	0.138 ± 0.009 b	ND	0.135 ± 0.010 b	ND	ND
Rutin	0.340 ± 0.005 b	0.339 ± 0.018 b	0.464 ± 0.132 ab	0.600 ± 0.168 a	0.39 ± 0.040 ab	0.390 ± 0.043 ab
Quercetin	0.281 ± 0.004 a	0.339 ± 0.049 a	0.283 ± 0.016 a	0.354 ± 0.085 a	0.287 ± 0.018 a	0.259 ± 0.014 a
Kaempferol	0.085 ± 0.008 b	0.098 ± 0.017 b	0.100 ± 0.014 b	0.086 ± 0.013 b	0.174 ± 0.038 a	0.104 ± 0.016 b
TOTAL	1.311 ± 0.013 cd	1.582 ± 0.063 bc	0.977 ± 0.136 d	1.309 ± 0.241 cd	2.194 ± 0.053 a	1.859 ± 0.084 b

ND represents compounds not detected through HPLC. ^1^ The different letters followed by the values in a column represents the significant difference (*p* < 0.005) between the parameter areas using Duncan’s multiple range test (n ≥ 3, mean ± SD).

**Table 4:** Datasets used for FT-IR and FT-NIR spectroscopy.

Technique (*n* = 600)	Samples	Number of Samples (Calibration)	Number of Samples (Prediction)
FT-IR spectroscopy	Arabidopsis powder samples	360	240

**Table 5:** Results from the developed PLSR, PCR, and HLA/GO models for the prediction of phenolic compounds in *A. thaliana* leaf powder samples using FT-IR spectroscopy.

Region	Model/Preprocessing	$R_{c}^{2}$	RMSEC (mg/g)	$R_{p}^{2}\;$	RMSEP (mg/g)	LVs
FT-IR spectroscopy	PLSR/Mean norm.	0.983	0.051	0.978	0.058	8
	PLSR/MSC	0.981	0.054	0.981	0.056	8
	PLSR/SNV	0.981	0.053	0.980	0.055	8
	PLSR/SG-1	0.969	0.053	0.975	0.063	8
	PLSR/SG-2	0.968	0.071	0.972	0.066	5
	PLSR/Raw	0.970	0.069	0.968	0.070	8
	PCR/SG-1	0.949	0.089	0.963	0.077	6
	HLA/GO/SNV	0.929	0.109	0.941	0.100	8

RMSEC, root mean square error of calibration; RMSEP, root mean square error of prediction.

**Table 6:** Results from the developed PLSR, PCR, and HLA/GO models developed for the prediction analysis of phenolic compounds in *A. thaliana* leaf powder samples using FT-NIR spectroscopy.

Region	Model/Preprocessing	$R_{c}^{2}$	RMSEC (mg/g)	$R_{p}^{2}$	RMSEP (mg/g)	LVs
FT-NIR spectroscopy	PLSR/Mean norm.	0.943	0.094	0.931	0.104	5
	PLSR/MSC	0.999	0.003	0.999	0.003	7
	PLSR/SNV	0.999	0.003	0.999	0.004	7
	PLSR/SG-1	0.993	0.031	0.991	0.036	6
	PLSR/SG-2	0.993	0.032	0.991	0.037	5
	PLSR/Raw	0.927	0.107	0.912	0.118	6
	PCR/MSC	0.999	0.004	0.999	0.003	6
	HLA/GO/SNV	0.922	0.116	0.897	0.131	5

## Data Availability

All the necessary data were contained in the paper.
